# *In Vivo* Imaging and Characterization of Actin Microridges

**DOI:** 10.1371/journal.pone.0115639

**Published:** 2015-01-28

**Authors:** Pui-ying Lam, Steve Mangos, Julie M. Green, Jochen Reiser, Anna Huttenlocher

**Affiliations:** 1 Department of Medical Microbiology and Immunology, University of Wisconsin-Madison, Madison, WI 53706, United States of America; 2 Program in Cellular and Molecular Biology, University of Wisconsin-Madison, Madison, WI 53706, United States of America; 3 Department of Internal Medicine, Rush University Medical Center, Chicago, IL 60612, United States of America; 4 Department of Pediatrics, University of Wisconsin-Madison, Madison, WI 53706, United States of America; Vanderbilt University Medical Center, UNITED STATES

## Abstract

Actin microridges form labyrinth like patterns on superficial epithelial cells across animal species. This highly organized assembly has been implicated in mucus retention and in the mechanical structure of mucosal surfaces, however the mechanisms that regulate actin microridges remain largely unknown. Here we characterize the composition and dynamics of actin microridges on the surface of zebrafish larvae using live imaging. Microridges contain phospho-tyrosine, cortactin and VASP, but not focal adhesion kinase. Time-lapse imaging reveals dynamic changes in the length and branching of microridges in intact animals. Transient perturbation of the microridge pattern occurs before cell division with rapid re-assembly during and after cytokinesis. Microridge assembly is maintained with constitutive activation of Rho or inhibition of myosin II activity. However, expression of dominant negative RhoA or Rac alters microridge organization, with an increase in distance between microridges. Latrunculin A treatment and photoconversion experiments suggest that the F-actin filaments are actively treadmilling in microridges. Accordingly, inhibition of Arp2/3 or PI3K signaling impairs microridge structure and length. Taken together, actin microridges in zebrafish represent a tractable *in vivo* model to probe pattern formation and dissect Arp2/3-mediated actin dynamics *in vivo*.

## Introduction

Superficial epithelial cells make labyrinth like patterns known as actin microridges. Microridges are observed on mucosal surfaces across animal species, including the human uterine epithelium [1], post-menopausal fallopian tubes [2], rat kidney collecting ducts [3], the cornea of multiple species [4,5,6]; and the surface epithelium of zebrafish larvae [7,8]. The ubiquitous presence and remarkable organization of microridges across animal species suggest an important function. It is generally thought that microridges help with mucus retention [9] and they have been hypothesized to provide rigidity and plasticity to surface epithelial cells [10,11]. However, the signaling pathways that regulate microridge structure remain largely unknown. Previous studies using transmission electron microscopy and atomic force microscopy of epithelial cells *in vitro*, have shown that microridges contain filamentous actin (F-actin) bundles [10,11,12]. Microridges form only in the most superficial layer of confluent epithelial cells *in vitro* [11] and are not observed in single epithelial cells [13,14] suggesting that the presence of a monolayer or cell-cell contact is essential for their formation. In addition, proper lipid secretion and lamellar granule trafficking have been suggested to regulate microridge structure [7]. However, the nature of F-actin in microridges remains unclear. Uehara et al. have previously shown by scanning electron microscopy that some epithelial cells are distended and have thicker microridges when treated with cytochalasin B [10]. Sharma et al., on the other hand, have shown by fluorescence microscopy and cytochalasin B treatment that F-actin in microridges is more stable than F-actin in lamellipodia [11]. Here we define the composition and dynamics of actin microridges using real time imaging in intact zebrafish (*Danio rerio*) larvae. We found that Arp2/3 and PI3K are necessary for microridge length, but myosin II activity is dispensable. Both Rac and Rho activity regulate the distance between microridges. This work provides new insights into how actin microridges are regulated *in vivo*, and provides a platform to analyze pattern formation in a dynamic Arp2/3 driven actin network in live animals.

## Results

### Microridge structure and composition

**Figure 1:**
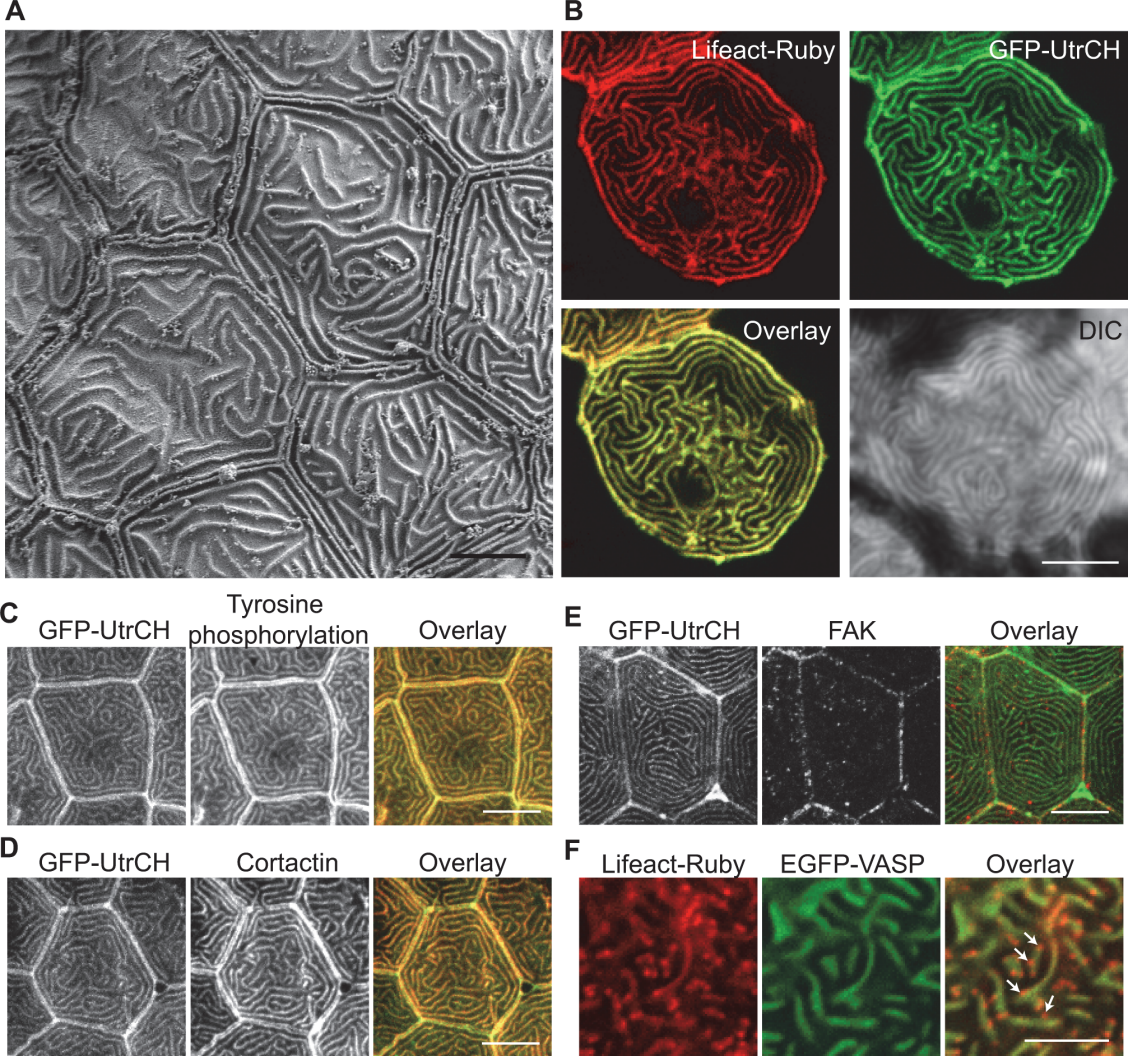
Microridge structure and composition. (A) Scanning electron microscopy on zebrafish larvae at 2 days post fertilization (dpf) in the area of the trunk. Scale bar, 5 μm. (B) Single plane confocal images of epithelial cells expressing actin bioprobes Lifeact-Ruby and GFP-UtrCH, which label all F-actin and stable F-actin, respectively. Signal from Lifeact-Ruby and GFP-UtrCH colocalize. Scale bar, 10 μm. (C-E) *Tg(krt4-GFP-UtrCH)* larvae at 2.5 dpf were fixed and immunostaining was performed using anti-phosphotyrosine antibody 4G10 (C), anti-cortactin antibody (D) or anti-FAK antibody (E). Scale bar, 10 μm. (F) Single plane confocal images of epithelial cells expressing actin bioprobe Lifeact-Ruby and EGFP-VASP. VASP localizes to a subset of Lifeact labeled microridges (S1 Movie). Arrows indicate regions of Lifeact labeling that did not colocalize with VASP. Scale bar, 5 μm.

To analyze microridge structure, we performed scanning electron microscopy (SEM) on zebrafish at different developmental stages and found that microridges appear as early as 12 hours post fertilization in epithelial cells that seem to have recently undergone cytokinesis (S1 Fig.). At this stage of development, however, most cells do not form microridges (S1 Fig.). By 1 day post fertilization (dpf), microridges are present on all epithelial surfaces examined, including the cornea, yolk and trunk region (Fig. 1A, S1 Fig.). Microridges generally become more prominent as larvae develop, with the exception of the cornea where microridges diminish by 4–5 dpf (S1 Fig.). Interestingly, microridges reappear on the cornea in adults (S1 Fig.).

Previous studies have shown that microridges contain F-actin bundles [10,11,12]. We were interested in testing if there is a polarity of F-actin dynamics in microridges. One example of such F-actin polarity has been reported in motile neutrophils in zebrafish larvae, where more dynamic F-actin is at the cell front and stabilized F-actin is at the uropod [15]. We expressed two F-actin binding probes specifically in epithelial cells of zebrafish larvae using the *krt4* promoter: Lifeact fused to Ruby, which detects all F-actin [16]; and the calponin homology domain of utrophin (UtrCH) fused to GFP, which detects more stable F-actin [17]. Confocal analysis showed that Lifeact and UtrCH labeled F-actin colocalize in microridges (Fig. 1B), suggesting that there is no obvious polarity of F-actin dynamics in microridges.

Focal adhesions (FAs) are well-studied actin containing structures on the cell cortex where F-actin bundles anchor and connect to the extracellular matrix. The formation of these F-actin bundles is tightly regulated in part by actin regulatory proteins that localize to FAs (reviewed in [18,19,20,21,22]. We next performed immunofluorescent staining and expressed fluorescently tagged proteins to identify the actin regulatory proteins that localize to microridges. We found that tyrosine phosphorylated proteins (Fig. 1C) and cortactin (Fig. 1D) colocalize with F-actin in microridges. By contrast, focal adhesion kinase (FAK) localizes to cell-cell contact sites but not to microridges (Fig. 1E). Vasodilator-stimulated phosphoprotein (VASP) is associated with FAs and is thought to have an important role in F-actin assembly [23]. Using fluorescently tagged VASP expressed in epithelial cells, we performed live dual imaging of VASP and Lifeact-Ruby labeled F-actin. VASP colocalizes with Lifeact at microridges. Interestingly, there are puncta labeled only with Lifeact but not VASP along the microridges and occasionally at the tips of filaments (Fig. 1F; S1 Movie). The localization of tyrosine phosphorylated proteins, cortactin and VASP, but not FAK, in microridges suggests that the highly organized F-actin bundles in microridges may have mechanisms of regulation that are similar to focal adhesions, stress fibers or other actin structures, like invadopodia.

### Microridge dynamics *in vivo*

**Figure 2:**
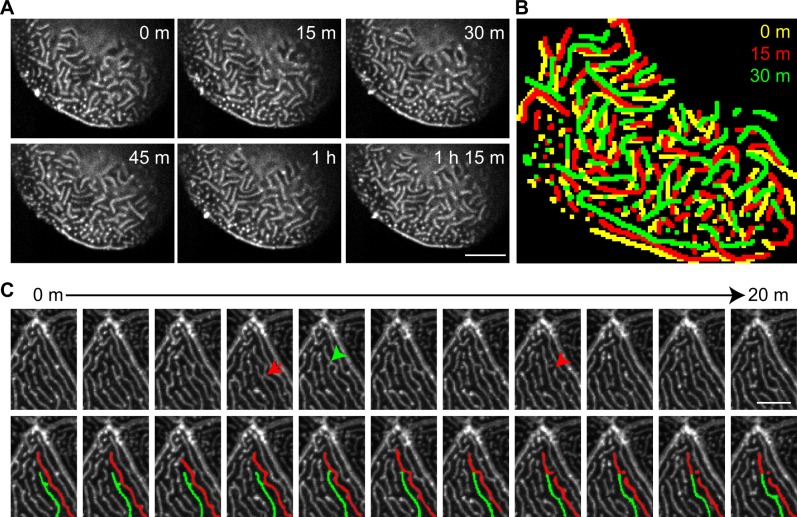
Microridges are dynamic *in vivo*. GFP-UtrCH was transiently expressed in epithelial cells in zebrafish larvae at 2.5 dpf. (A) Maximum intensity projection of confocal images showing changes in microridge pattern over time (S2 Movie). Scale bar, 10 μm. (B) Overlay of microridge pattern in the same cell at time points indicated. Microridges are color-coated to indicate patterns from different time points. (C) Time-lapse maximum intensity projection images showing how microridge patterns rapidly change by bending (red arrow), breaking (red arrowhead) and connecting (green arrowhead) (S3 Movie). (Top panel) Images acquired every 2 minutes are shown. (Bottom panel) Duplication of the top panel with two adjacent microridges labeled with pseudo-color to aid in the visualization of microridge dynamics. Scale bar, 5 μm.

Microridges form highly organized patterns *in vivo*. We next wanted to determine if these organized F-actin structures are dynamic using real time imaging of F-actin bioprobes in intact animals. A previous study using phase contrast imaging on keratocytes *in vitro* revealed that microridges are dynamic within monolayers [11]. However, the keratocytes in monolayers are also mobile [11], while epithelial cells in zebrafish larvae are relatively stationary. To achieve fast, high-resolution image acquisition of microridges within a three-dimensional live animal, we expressed GFP-UtrCH specifically in epithelial cells in zebrafish larvae to visualize F-actin in microridges. Confocal time-lapse imaging showed that microridges are constantly shifting in position through bending, merging and breaking (Fig. 2A-2C; S2 and S3 Movies), consistent with *in vitro* observations [11]. These findings suggest that although microridges maintain a highly ordered structure, microridges are dynamic *in vivo*.

**Figure 3:**
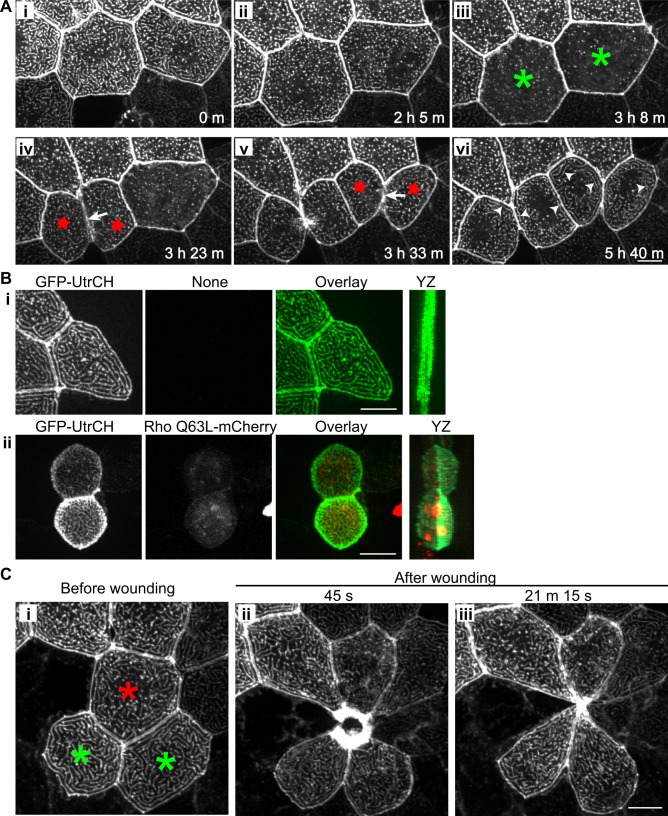
Microridge organization during cytokinesis and wounding. (A) Time-lapse maximum intensity projection images show changes in microridge pattern during cytokinesis at 30 hours post fertilization (S4 Movie). Mosaic transient expression of GFP-UtrCH in epithelial cells allows visualization of microridges. Microridges gradually became shorter before cytokinesis (i and ii). Microridges transiently disappear (iii; in cells labeled with green asterisk) shortly before the appearance of the cleavage furrow (iv and v; arrows); reappear quickly during cleavage furrow formation (iv and v; in cells labeled with red stars); and then gradually become longer after cytokinesis (vi; arrowheads). Data are representative of 6 individual cells from 3 zebrafish larvae. Scale bar, 10 μm. (B) Maximum intensity projection of epithelial cells expressing GFP-UtrCH alone (i) or with constitutive active Rho (Rho Q63L-mCherry; ii) in zebrafish larvae at 2.5 dpf. Active Rho expression in epithelial cells results in cell rounding and apical protrusion but microridges are maintained. YZ, maximum intensity projection on the YZ axis. Data are representative of 63 individual cells in 21 larvae from three separate experiments. Scale bar, 10 μm. (C) Rearrangement of microridges during epithelial wound healing *in vivo*. Laser cell ablation was performed on a single cell (red asterisk) in a zebrafish larva at 2.5 dpf that transiently expresses GFP-UtrCH in epithelial cells. Maximum intensity projection images before (i) and after laser ablation (ii and iii) show the change in microridge pattern and cell shape in the epithelial cells (green asterisk) adjacent to the wound (S5 Movie). Data are representative of 5 individual experiments. Scale bar, 10 μm.

To determine if cell perturbations alter microridge structure, we visualized actin microridges during cytokinesis and after wounding using confocal live imaging of epithelial cells expressing GFP-UtrCH. Interestingly, microridges are reduced to sparse, small foci of actin prior to cytokinesis (Fig. 3A; S4 Movie). Before the appearance of the cleavage furrow, microridges are shorter and more punctate (Fig. 3Ai, ii), and then gradually diminish with a near homogeneous distribution of GFP-UtrCH right before the cleavage furrow forms (Fig. 3Aiii). During cleavage furrow formation, microridges first reappear as dots (Fig. 3Aiv, v) and then progress to form longer structures as cleavage furrow formation progresses. Shortly after cytokinesis, F-actin re-assembles to form the organized microridge pattern (Fig. 3Avi). The neighboring epithelial cells also undergo changes in cell shape without the disappearance of microridges. It is interesting that microridges on superficial epithelial cells are constantly changing although the overall pattern is maintained over time.

Activation of Rho is essential during cytokinesis in eukaryotic cells and is a key regulator of the actin cytoskeleton (reviewed in [24]). We therefore tested if an increase in Rho activity was sufficient to alter microridge structure by expressing constitutively active Rho (Rho Q63L) in epithelial cells of zebrafish larvae. Epithelial cells on zebrafish larvae are usually polygonal and flat (Fig. 3Bi). Rho Q63L expression induces cell rounding and apical protrusions from the cell surface (Fig. 3Bii). However, microridges are still present despite the change in cell shape, even with cell rounding, suggesting that the F-actin bundles are likely associated with the cortical actin network. Taken together, these findings suggest that an increase in Rho activity and cell shape change alone is not a significant determinant of microridge architecture.

We next determined if microridges change during wound closure. Multicellular epithelial wound closure in embryos is often mediated by the formation of a contractile actomyosin purse string at wound edges [25,26,27], which requires Rho activation [25,27]. To test how wounding alters microridge structure, we performed single cell laser ablation on zebrafish larvae that express GFP-UtrCH in epithelial cells (Fig. 3Ci). Confocal live imaging showed that microridges are present in the responding epithelial cells during cell contraction and wound closure (Fig. 3Cii, iii). The presence of microridges following wounding further confirms the finding that an increase in Rho activity is not sufficient to induce microridge disassembly. However, with wounding we observed rearrangement in the orientation of microridges at the wound edge. Live imaging revealed that microridges bend, break and rearrange after the laser-induced wounding, but maintain their overall structure (Fig. 3C; S5 Movie). Further investigation will be required to address whether the change in microridge orientation in these responding epithelial cells is due to changes in cell signaling or is due to the physical changes involved in wound closure. Taken together, these findings suggest that the overall structure of microridges may be relatively independent of changes in cellular tension, in contrast to focal adhesions and stress fibers.

### Regulation of microridge architecture

**Figure 4:**
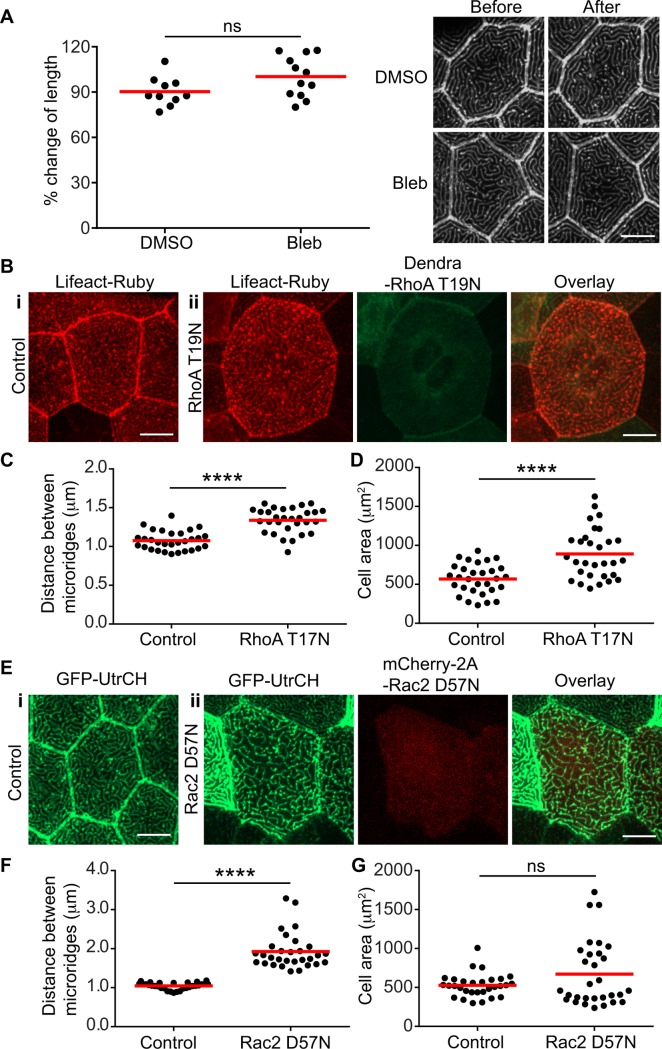
Rho GTPases regulate microridge organization. (A) Larvae at 2.5 dpf were treated with 25 μM Blebbistatin (Bleb) for 1 h to inhibit myosin II. 0.5% DMSO was used as a control. The length of microridges in the same cell before and after treatment was measured. Each data point represents the percentage change of the average microridge length in an individual cell after treatment. Data are representative of at least three different experiments. Each experiment was performed on at least 3 larvae, with measurements performed on 3–4 cells per larva. For details of the methods and analysis, refer to the Methods and Materials section. Efficacy of blebbistatin was confirmed by changes in neutrophil morphology and motility after treatment (data not shown). ns, not significant (two-tailed, unpaired *t*-test). (Right panel) Representative confocal images of GFP-UtrCH labeled microridges before and after treatment. Scale bar, 10 μm. (B) Maximum intensity projection of epithelial cells expressing Lifeact-Ruby alone (i) or with dominant negative RhoA (Dendra2-RhoA T19N) (ii) in zebrafish larvae at 2.5 dpf. Scale bar, 10 μm. Average distance between microridges (C) and cell area (D) in control or Dendra2-RhoA T19N expressing epithelial cells were measured and compared. Each data point represents the value from one cell. Data are collected from more than ten individual larva for each group from 3 separate experiments. **** *P*<0.0001 (two-tailed, unpaired *t*-test). (E) Maximum intensity projection of epithelial cells expressing GFP-UtrCH alone (i) or with dominant negative Rac2 (mCherry-2A-Rac2 D57N) (ii) in zebrafish larvae at 2.5 dpf. Scale bar, 10 μm. Average distance between microridges (F) and cell area (G) in control or mCherry-2A-Rac2 D57N expressing epithelial cells were measured and compared. Each data point represents the value from one cell. Data are collected from more than twelve individual larva for each group from 3 separate experiments. For details of the methods and analysis refer to the Methods and Materials section. **** *P*<0.0001; ns, not significant (two-tailed, unpaired *t*-test).

To further investigate the role of motors and cellular tension on microridges, we tested the effects of myosin II inhibition. Previous studies have supported an essential role for myosin II activity in stress fiber formation and the regulation of focal adhesions (reviewed in [19]). To determine the role of myosin II in microridges, we analyzed microridge length in the same cell from intact zebrafish larvae before and after treatment with blebbistatin. Measuring microridge length in the same cell helped to control for the variation in microridges between different cells. Myosin II inhibition has no effect on the length or overall organization of microridges compared to control treatment (Fig. 4A). To further test the possible role of myosin activity in microridges, we performed immunofluorescent staining using an antibody that recognizes phospho-myosin light chain (pMLC). pMLC is detected in muscle fibers under the epithelium as expected (data not shown), but surprisingly, is not detected in microridges (S2 Fig.). These findings suggest that myosin II activity is dispensable for the maintenance of microridges.

To further probe the role of actomyosin contractility in microridge structure, we expressed dominant negative RhoA (RhoA T19N) in epithelial cells. We found that microridges are still present in cells expressing RhoA T19N (Fig. 4B). However, there is a wider distance between these microridges (Fig. 4C), which correlates with an increase in cell area (Fig. 4D). One caveat is that we could not measure microridge length in the same cell before and after Rho inhibition, since RhoA T19N is driven constitutively from the *krt4* promoter. Further experiments will be needed to address how RhoA modulates cell size and the distance between microridges. Rac is another small Rho GTPase that regulates actin polymerization. We tested the involvement of Rac in microridge architecture by expressing a dominant negative form of Rac (Rac2 D57N), which inhibits both endogenous Rac1 and Rac2 [28], in epithelial cells using the *krt4* promoter. Microridges persist even with the constitutive expression of Rac2 D57N (Fig. 4E). However, we observed an increase in the distance between microridges (Fig. 4F) with no significant change in the average cell size (Fig. 4G). Our data suggest that both Rho and Rac are involved in the regulation of microridge organization.

### F-actin is treadmilling in microridges

**Figure 5:**
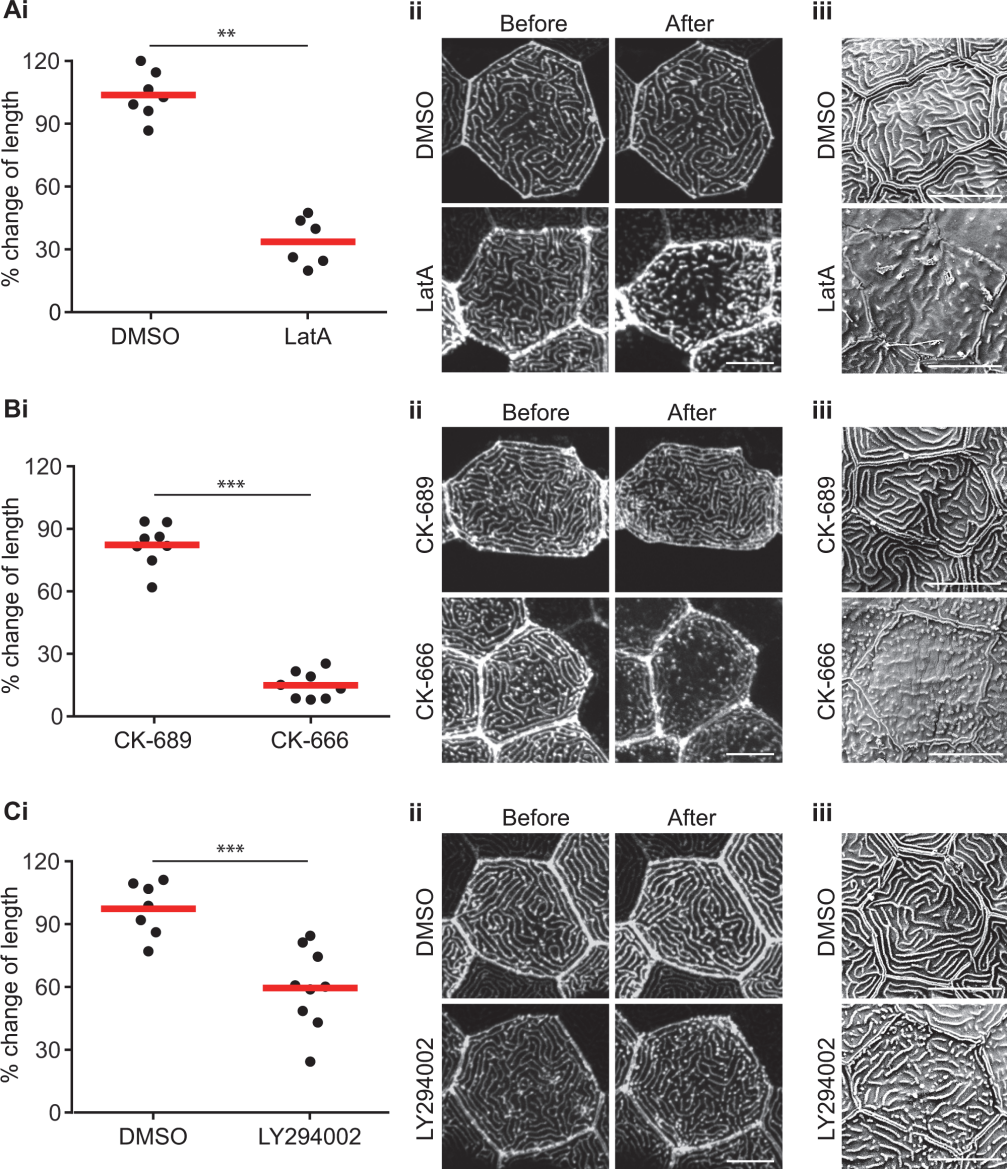
Microridges are actively treadmilling. Inhibition of actin polymerization using Latrunculin A (LatA) (A) or inhibition of Arp2/3 using CK-666 (B) resulted in shortened microridges. Larvae at 2.5 dpf were treated with 1 μM LatA or 0.5% DMSO as controls for 30 minutes, or with 200 μM CK-666 or its inactive analogue CK-689 for 1 hour. (C) PI3K kinase activity is involved in maintaining microridge length. Larvae at 2.5 dpf were treated with 130 μM LY294002 for 1 hour to inhibit PI3K. 0.5% DMSO was used as a control. (Ai, Bi and Ci) The length of microridges in the same cell before and after treatment was measured and compared. Each data point represents the percentage change of average microridge length in one cell after treatment. Data are representative of at least three separate experiments. Each experiment was performed on at least 3 larvae, with microridge measurements on 3–4 cells per larva. For details of the methods and analysis refer to the Methods and Materials section. ** *P*<0.01; *** *P*<0.001 (two-tailed, unpaired *t*-test). (Aii, Bii and Cii) Representative confocal images of GFP-UtrCH labeled microridges before and after the respective treatments. Scale bar, 10 μm. (Aiii, Biii and Ciii) Representative scanning electron microscopy images of zebrafish larvae with the respective treatments. Scale bar, 10 μm

It is unclear how microridges are both highly dynamic yet maintain an intrinsic organization that remains stable, even after perturbations like wounding. In order to study F-actin dynamic in microridges, we inhibited F-actin polymerization using Latrunculin A (LatA). Microridge length in the same cell before and after treatment was visualized, measured and quantified using GFP-UtrCH localization and confocal microscopy. LatA treatment results in shorter microridges and fewer microridges as compared to DMSO controls (Fig. 5Ai-ii). The surface topology of this microridge phenotype was further confirmed using SEM (Fig. 5Aiii). In contrast to *in vitro* studies [11], these findings suggest that F-actin in microridges is actively treadmilling and that actin polymerization is required to maintain microridge structure. Our findings are consistent with an earlier *in vivo* study using carp epithelium [10].

Previously reported transmission electron microscopy images of carp epithelial microridges show branching of F-actin filaments in microridges [10]. This is reminiscent of Arp2/3 mediated actin assemblies *in vitro*. To determine if Arp2/3 regulates microridges, Arp2/3 was inhibited using the small molecule CK-666. We found a dramatic change in microridge architecture in the presence of Arp2/3 inhibition, with substantially shorter microridges compared to control inhibitor (Fig. 5Bi-ii). Similar to the LatA experiment, SEM analysis confirmed that the surface topology closely resembles the underlying actin microridge pattern (Fig. 5Biii). To further identify signaling pathways that regulate microridge structure, we tested the effects of PI3K inhibition, since PI3K has been implicated in dynamic F-actin at the leading edge of motile cells *in vivo* [15]. PI3K inhibition using LY294002 impairs microridge length compared to control (Fig. 5Ci-iii). Our results suggest that F-actin in microridges is actively treadmilling and that Arp2/3 and PI3K regulate actin microridge structure.

**Figure 6:**
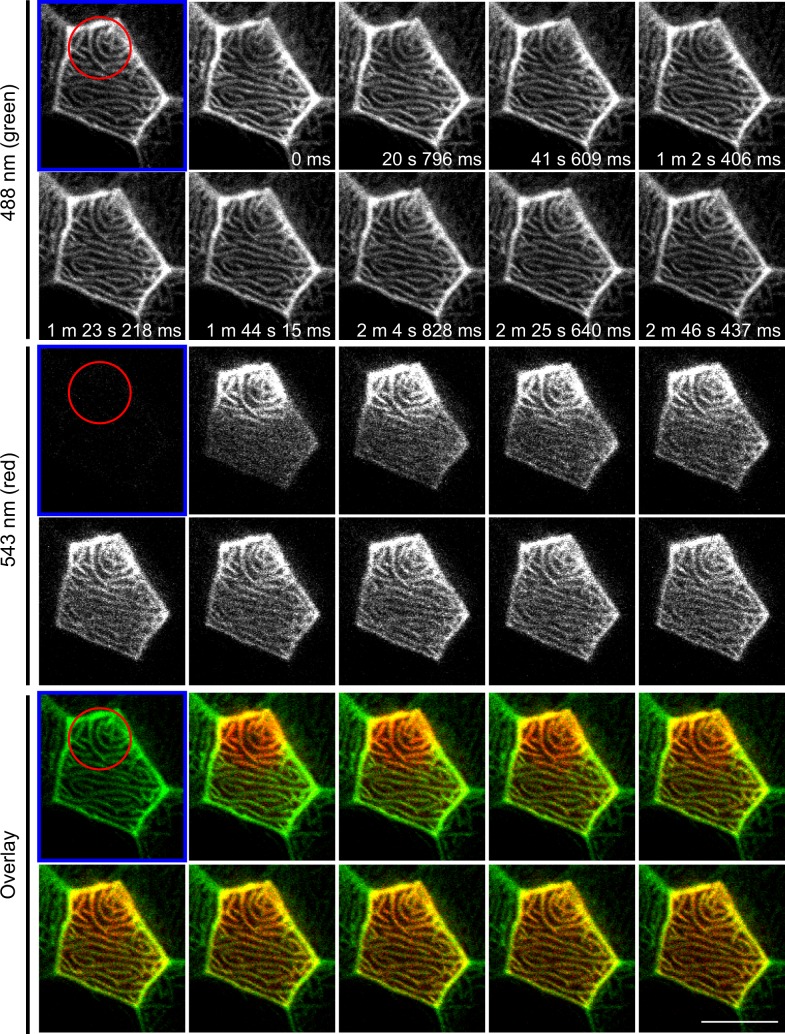
F-actin in microridges is highly dynamic. Photoconversion of actin-mEos was performed on 3 dpf larvae by focusing on a sub-region (red circle) of an epithelial cell displaying microridges *in vivo* and time-lapse images were taken before (labeled with blue frame) and after photoconversion. Upon photoconversion, mEos-actin is irreversibly converted from emitting a green to a red fluorescent signal. For details of photoconversion refer to Materials and Methods. Single plane of confocal image of red channel, green channel and overlay of the two are shown. Data are representative of 9 individual cells. Scale bar, 10 μm. See S7 Movie.

To further test the dynamics of F-actin in microridges we examined the turnover of mEos-tagged actin after photoconversion in epithelial cells *in vivo*. mEos is irreversibly converted from green to red fluorescent emission upon 405 nm laser excitation. A small region of the microridge was photoconverted and time-lapse images were acquired after photoconversion. The signal from the photo-converted actin in the microridge rapidly diffuses and weakens after a few minutes (S3 Fig., S6 Movie). This suggests that actin is actively depolymerizating in microridges. When a larger region of the microridge was photoconverted, the non-converted microridge region shows incorporation of photoconverted actin within a few minutes (Fig. 6, S7 Movie). As photoconversion also occurs in the pool of un-incorporated actin-mEos in the cytosol, this observation suggests that the incorporation of actin into microridges is very rapid. As a control, mEos alone was expressed in epithelial cells. Under these conditions, no microridge pattern was observed (S4 Fig.), confirming the specificity of the actin-mEos photoconversion in microridges. Overall, our findings suggest that F-actin in microridges is highly dynamic.

## Discussion

Microridges have been observed on epithelial cells of the mucosal epithelium across animal species from fish to humans. The mechanisms that regulate the formation and maintenance of microridges remain largely unknown. Here for the first time, we characterize the dynamics of actin microridges in intact animals using live imaging. We found that actin microridges form a dynamic Arp2/3-dependent F-actin network in superficial epithelial cells of live zebrafish that is amenable to high-resolution live imaging. We also found that inhibition of myosin II does not significantly affect actin microridge length. Moreover, expression of dominant negative RhoA or Rac affects the distance between microridges but not their overall organization. These findings, together with the observation that microridges bend *in vivo*, suggest that significant tension is not necessary to maintain their structure. As proper actin polymerization is essential for the microridge structure, we speculate that other actin regulators are likely involved in the formation and maintenance of actin microridges.

The reduction of microridges that occurs before cytokinesis is intriguing. It has been proposed that cell protrusions can serve as a membrane reservoir for other cellular processes (reviewed in [29]). One example is during *Drosophila* cellularization, where microvilli flatten out during cleavage furrow ingression to fuel cell-surface growth [30]. Unlike microvilli during *Drosophila* cellularization, it is not clear whether microridges provide a membrane reservoir during epithelial cell cytokinesis. Microridges shorten and become drastically reduced before an obvious cleavage furrow is formed and before a change in cell shape is observed during cytokinesis. This suggests that the disappearance of microridges precedes the change in membrane tension generated by the cleavage furrow and the need for extra plasma membrane. Interestingly, the reappearance of microrridges and actin puncta coincides with cleavage furrow ingression. This further suggests that microridge formation can occur in the presence of high cell membrane tension. These observations suggest that in addition to positive signaling through Arp2/3 and PI3K, there are likely inhibitory signaling pathways that negatively regulate microridge structure. Understanding how microridges change during cytokinesis should provide valuable insight into the regulation of microridge architecture.

It is interesting that the change in membrane tension and cell shape that occurs during wound closure affects microridge arrangement but not its overall structure. To our knowledge, microridge rearrangement has not been described previously in other wound healing models, but this may be due to the difference in model systems [25,26,27,31,32]. Since both wound closure and cytokinesis require localized actin polymerization, the maintenance of microridges during these processes suggests that microridges do not simply function as a reservoir for actin. As microridges are ubiquitously present on mucosal surfaces across species, further investigation will be needed to understand microridge rearrangements on different epithelial surfaces following wounding. In addition, we have observed that microridges on the cornea diminish starting at 4–5 dpf (S1 Fig.) but are then re-established in adults (S1 Fig.). This has not previously been reported during cornea development [33], and will provide an additional area for future investigation.

Alan Turing proposed that organized patterns, like microridges, arise from an interaction between two molecules that diffuse at different rates [34]. This reaction-diffusion model or ‘Turing model’ mathematically predicts how spatial patterns develop autonomously and has been used as a framework for understanding biological pattern formation (reviewed in [35]). Using Turing theory, the interaction between two morphogens, such as an activator and an inhibitor, can result in a labyrinth of color patterns [36] that appears to be very similar to the microridge pattern in epithelial cells. Analogous to the unique nature of markings seen on animals within a species with respect to the exact position of each spot or stripe, microridge patterns appear to be different among individual epithelial cells within the same animal. In addition, the overall microridge pattern is distinguishable across species [1,2,3,4,5,6,7,8,37,38,39,40,41,42]. However, unlike a stationary animal color pattern, the change in microridge pattern over time suggests a dynamic change in morphogen-like interactions within individual cells. We speculate that the subcellular pattern of microridges might be another biological example of the Turing mechanism. In this study, we have found that Arp2/3 and PI3K activity are positive regulators of microridge length. Rac and Rho both play a role in establishing the distance between microridges with the caveat that Rho activity also affects cell size. The molecular events involved in forming microridges, including the diffusion efficiency of the above listed regulators, their interactions, and other yet-identified activators/inhibitors requires further exploration.

In summary, our findings have provided new insights into the dynamics and regulation of microridges *in vivo*. This work may open up exciting avenues for probing the regulation of Arp2/3 dependent actin networks and pattern formation in an intact animal. It also provides a physiologically relevant context for investigating the regulation and function of microridges. A challenge for future investigation will be to use this system to test the “Turing” model in an *in vivo* tissue to uncover the elaborate signaling networks needed to maintain these beautiful actin-based structures present on surface epithelia.

## Materials and Methods

### Ethics Statement

This study was carried out in strict accordance with the recommendations in the Guide for the Care and Use of Laboratory Animals of the National Institutes of Health. The protocol was approved by the University of Wisconsin—Madison Animal Care and Use Committee (Protocol number: M01570-0-02-13).

### Zebrafish lines

Wild-type AB zebrafish (*Danio rerio*) were maintained and raised, as described previously [43]. Embryos were kept at 28.5°C in E3 solution.

### DNA injection

All DNA expression vectors contained the zebrafish *krt4* promoter for epithelial cell expression [44,45]. All expression vectors contain minimal Tol2 elements for efficient integration [46] and an SV40 polyadenylation sequence (Clontech Laboratories, Inc). The following constructs were generated: *krt4-Lifeact-Ruby, krt4-GFP-UtrCH, krt4-EGFP-VASP* (XM_005173679.1), *krt4-Rho Q63L-mCherry* [15], *krt4-Dendra2-RhoA T19N* [15], *krt4-mCherry-2A-Rac2 D57N* [28], *krt4-mEos* and *krt4-actin-mEos* [47]. Expression of constructs was obtained by injecting 3 nL of solution containing 3–3.6 ng/μL of DNA plasmid and 8.7 ng/μL of *in vitro* transcribed (Ambion) Tol2 transposase mRNA into the cytoplasm of one-cell stage embryos.

### Scanning Electron Microscopy

Zebrafish larvae or dissected adult eyes were fixed at the indicated times in EM fix (1.5% glutaraldehyde; 1% paraformaldehyde; 70 mM NaPO_4_; 3% sucrose). Specimen were then rinsed in 0.1 M Cacodylate buffer, postfixed in 1% OsO_4_, rinsed again in 0.1 M Cacodylate buffer then dehydrated in a graded series of ethanol dilutions to 100% ethanol. Samples were then critically dried using liquid CO_2_ as the substitution medium (EMS 850 Critical Point Dryer), then adhered to aluminum specimen mounts (Ted Pella) using carbon conductive cement (Plano GMBH). A Cressington 108 auto Sputter Coater (Ted Pella) was used to gold coat the samples. Imaging was carried out using a Zeiss Sigma HDVP Electron Microscope.

### Immunohistochemistry and imaging

2.5 dpf larvae were fixed with 1.5% formaldehyde in 0.1 M Pipes, 1 mM MgSO_4_, and 2 mM EGTA overnight at 4°C. Larvae were then washed in PBS and incubated in methanol at −20°C overnight. Larvae were rehydrated in a graded series of methanol in PBS containing 0.2% Triton X-100 (PBSTx) and incubated with 0.15 M glycine in PBS for 10 min at room temperature. Larvae were then washed with PBSTx several time and blocked in PBSTx containing 1% BSA for 1 hour at room temperature and then with primary antibody at 4°C overnight. Following several washes in PBSTx, larvae were incubated with secondary antibody at 4°C overnight. We used the following primary antibodies: mouse anti-phosphotyrosine antibody, clone 4G10 (EMD Millipore 05–321) at 1:50 dilution; mouse anti-cortactin antibody, clone 4F11 (Millipore 05–180) at 1:100 dilution; rabbit anti-FAK (Cell Signaling #3285) at 1:50 dilution; rabbit anti-Myosin Light Chain Antibody, phospho-specific (Ser19) (EMD Millipore AB3381) at 1:50 dilution. For secondary antibody, anti-rabbit or anti-mouse DyLight 549-conjugated IgG antibodies (Jackson ImmunoResearch Laboratories, Inc.) at 1:250 dilution was used.

All images were acquired using a spinning disk confocal microscope (Yokogawa CSU-X) with a confocal scanhead on a Zeiss Observer Z.1 inverted microscope (NA1.3/60X water immersion objective) at 28.5°C. A Photometrics Evolve EMCCD camera was used to acquire the images. Z-series images were acquired using a 0.4 μM step size and 300 EM gain. Maximum intensity projection images were made using the Zen 2012 (blue edition) software (Carl Zeiss). For imaging before and after pharmacological treatments, larvae were anesthetized with 0.2 mg/mL tricaine in E3 and placed on a glass-bottom dish for imaging. For time-lapse imaging, larvae were mounted on a glass-bottom dish with 1% low-melting point agarose and covered with E3 containing tricaine. Carl Zeiss Immersol W 2010 immersion medium was used to reduce evaporation of the immersion medium and allow uninterrupted long-term imaging.

### Laser ablation and photoconversion

Larvae at 2.5 dpf were mounted on a glass-bottom dish with 1% low-melting point agarose. A Z-stack image of the targeted ablation and surrounding area in the trunk was acquired using the spinning disk confocal system before laser ablation. Laser ablation was performed using a laser scanning confocal microscope (FluoView FV1000, Olympus) with a NA1.3/40X oil objective. A 405 nm diode laser at maximal power was used, focusing on a small circular area of an epithelial cell (diameter ∼1 μm) for 3–4 minutes. Larvae were then transferred to the spinning disk system immediately for Z-stack time-lapse image acquisition. Photoconversion of actin-mEos was performed using the same microscope setup as laser ablation, except that the 405 nm laser was set at 3%, focusing on an area as indicated for 2 seconds. Images were acquired on the same Olympus laser scanning confocal microscope before and after photoconversion.

### Pharmacological treatments and microridge measurements

All pharmacological treatments were performed on zebrafish larvae at 2.5 dpf. 0.5% DMSO was used as controls except for CK-666 treatment. To inhibit myosin II, a 25 mM stock of Blebbistatin (Sigma B0560) was made in DMSO. Larvae were treated with 25 μM Blebbistatin (Bleb) for 1 hour. To inhibit actin polymerization, a 10 mM Latrunculin A (Fisher Scientific NC9114335) stock was made in DMSO. Larvae were treated with 1 μM Latrunculin A for 30 minutes. To inhibit Arp2/3, a 100 mM stock of CK-666 (Sigma SML0006) was made in DMSO. Larvae were treated with 200 μM CK-666 for 1 hour. 200 μM CK-689 (EMD Millipore 182517) used as an inactive control of CK-666. To inhibit PI3K, a 65 mM stock of LY294002 (EMD Millipore 440202) was made in DMSO. Larvae were treated with 130 μM LY294002 for 1 hour.

Measurement of microridge length: GFP-UtrCH was transiently expressed in epithelial cells to allow for clear visualization of microridges. Confocal Z-stack images of the same cell in the lateral side of the mid-trunk region were taken before and after pharmacological or control treatments. Maximum intensity projection images were generated using the Zen 2012 (blue edition) software (Carl Zeiss). The length of microridges in the same cell before and after treatments was measured using FIJI “Freehand Line” tool. 8–15 microridge length measurements were made per cell. Percentage change of length was calculated by dividing the average length of microridges in the same cell after treatment to that before treatment. All the inhibitor treatments were performed at least 3 separate times. Each experiment was performed on at least 3 larvae, with microridge measurements on 3–4 cells per larva.

Distance between microridges was measured manually using FIJI “Straight Line” tool. Lifeact-Ruby or GFP-UtrCH was transiently expressed in the epithelial cells of zebrafish larvae to allow for clear visualization of microridges. Confocal images were acquired at 2.5 dpf. To calculate the average distance between microridges, ten random measurements of the distance between two adjacent microridges were made per cell. Area of epithelial cells was measured manually using FIJI “Freehand selections” tool.

### Statistics

Experimental results were analyzed with Prism (GraphPad Software) statistical analysis software. The resulting *P* values are included in the figure legends for each experiment.

## Supporting Information

S1 Fig Microridge structure during zebrafish development. (Ai—Aii) Scanning electron microscopy images on zebrafish embryo at 12 hour post fertilization (hpf). (Ai) Microridges were observed in epithelial cells that appeared to have recently undergone cytokinesis. Arrows indicate example of microridges. (B) SEM images at 1–5 and 9 day post fertilization (dpf) on the cornea, yolk and trunk region of zebrafish larvae. (C) SEM images of cornea at adult stage. Scale bar, 5 μm.
(TIF)

S2 Fig Phospho-Myosin Light Chain antibody staining. Maximum intensity projection of confocal images show phospho-Myosin Light Chain (pMLC) antibody staining in *Tg(krt4:GFP-UtrCH)* larvae at 2.5 dpf. Scale bar, 10 μm.
(EPS)

S3 Fig F-actin in microridges is highly dynamic. Photoconversion of actin-mEos was performed on 3 dpf larvae by focusing on a small region of an epithelial cell containing microridges (red circle) and acquiring time-lapse images before (labeled with blue frame) and after photoconversion. Upon photoconversion, mEos-actin is irreversibly converted from exhibiting green to red fluorescent emission. Data are representative of 15 individual cells. For details of photoconversion refer to Materials and Methods. Single plane of confocal image of red channel, green channel and overlay of the two are shown. Scale bar, 10 μm. See S6 Movie.
(EPS)

S4 Fig mEos expression in epithelial cells *in vivo*. Krt4-mEos was transiently expressed in epithelial cell. Single Z-plane of DIC or mEos at 1.9 μm step size was shown. Scale bar, 10 μm.
(EPS)

S1 Movie Live imaging of VASP and F-actin *in vivo*. Lifeact-Ruby (left panel) and EGFP-VASP (middle panel) were transiently expressed in epithelial cells in zebrafish larvae at 2.5 dpf. (Right panel) Overlay of Lifeact-Ruby and EGFP-VASP. Images were acquired using a spinning disk confocal microscope. Images were taken every 15 seconds for 14 minutes and 45 seconds.
(MOV)

S2 Movie Microridges are dynamic *in vivo*. GFP-UtrCH was transiently expressed in epithelial cells in zebrafish larvae at 2.5 dpf. Images were acquired using a spinning disk confocal microscope. Images were taken every 15 minutes for 1 hour 45 minutes.
(MOV)

S3 Movie Microridges are dynamic *in vivo*. GFP-UtrCH was transiently expressed in epithelial cells in zebrafish larvae at 2.5 dpf. Images were acquired using a spinning disk confocal microscope. Images were taken every 1 minute for 20 minutes.
(MOV)

S4 Movie Change in microridge pattern during cytokinesis. GFP-UtrCH was transiently expressed in epithelial cells in zebrafish larvae at 2 dpf. Images were acquired using a spinning disk confocal microscope. Images were taken every 1 minute for 5 hours 46 minutes.
(MOV)

S5 Movie Change in microridge pattern during wound healing. GFP-UtrCH was transiently expressed in epithelial cells in zebrafish larvae at 2.5 dpf. Laser cell ablation was performed in a single cell (red asterisk) of zebrafish larvae transiently expressing GFP-UtrCH in epithelial cells. Images were acquired before and after laser wounding using a spinning disk confocal microscope. Images were taken every 15 seconds for 21 minutes.
(MOV)

S6 Movie Photoconversion of actin-mEos in microridges. Actin-mEos was transiently expressed in epithelial cells in zebrafish larvae at 3 dpf. Photoconversion of actin-mEos was performed in a small region (red circle) of an epithelial cell displaying microridges *in vivo*. Time-lapse images were taken before and after photoconversion. Left panel, signal of actin-mEos before photoconversion; middle panel, signal of actin-mEos after photoconversion; right panel, overlay of the two channels. Images were taken every 21 seconds for 9 minutes 42 seconds.
(MOV)

S7 Movie Photoconversion of actin-mEos in microridges. Actin-mEos was transiently expressed in epithelial cells in zebrafish larvae at 3 dpf. Photoconversion of actin-mEos was performed in a region (red circle) of an epithelial cell displaying microridges *in vivo*. Time-lapse images were taken before and after photoconversion. Left panel, signal of actin-mEos before photoconversion; middle panel, signal of actin-mEos after photoconversion; right panel, overlay of the two channels. Images were taken every 21 seconds for 9 minutes 42 seconds.
(MOV)
