# Supplementary figures and images for: In Vivo Imaging and Characterization of Actin Microridges
Source: PLoS One. 2015 Jan 28;10(1):e0115639. doi: 10.1371/journal.pone.0115639 (PMC4309568; doi:10.1371/journal.pone.0115639)

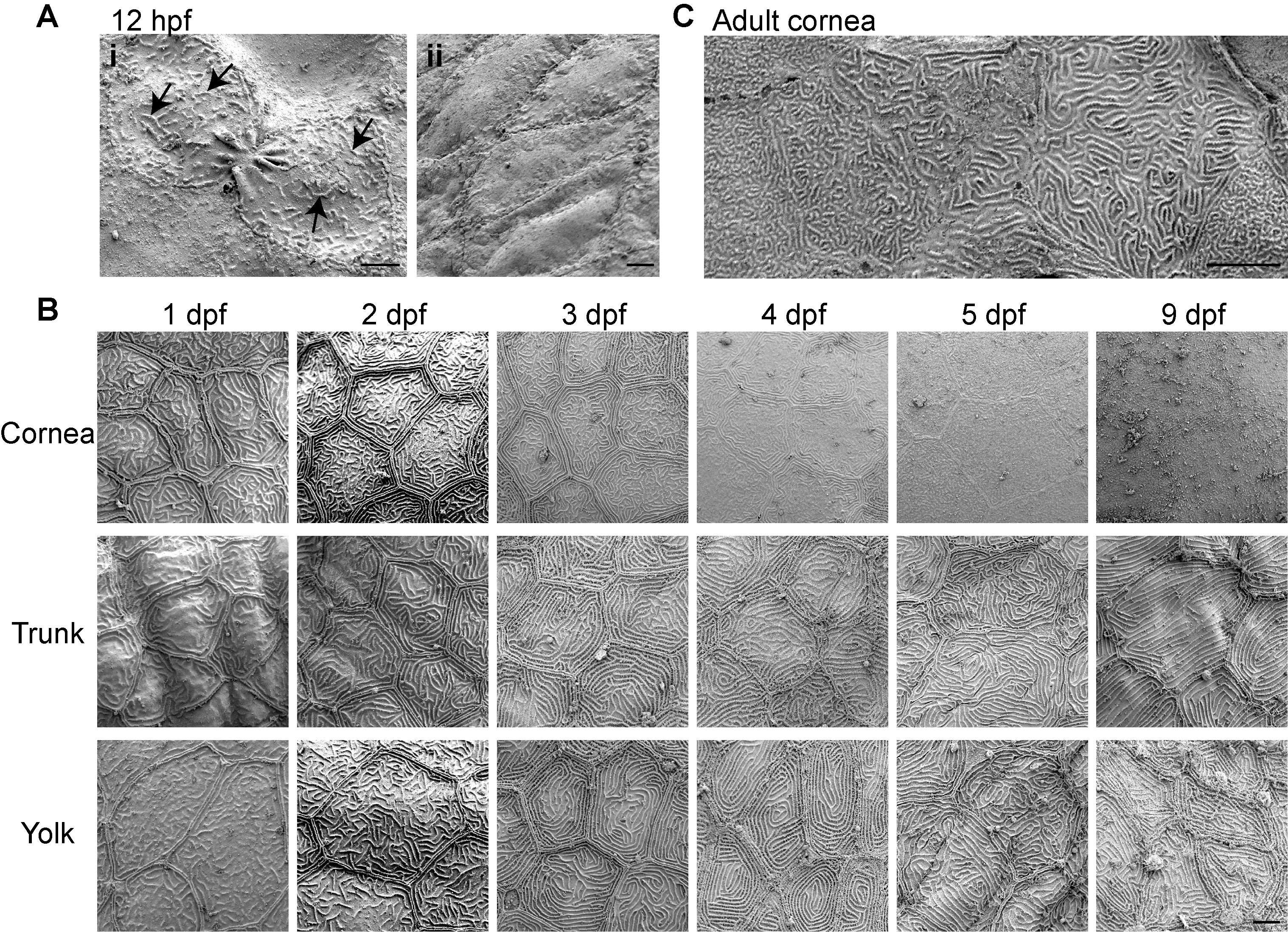

Supplement: S1 Fig — (Ai—Aii) Scanning electron microscopy images on zebrafish embryo at 12 hour post fertilization (hpf). (Ai) Microridges were observed in epithelial cells that appeared to have recently undergone cytokinesis. Arrows indicate example of microridges. (B) SEM images at 1–5 and 9 day post fertilization (dpf) on the cornea, yolk and trunk region of zebrafish larvae. (C) SEM images of cornea at adult stage. Scale bar, 5 μm. (TIF) [file pone.0115639.s001.tif]
